# The two-stage therapeutic effect of posture biofeedback training on back pain and the associated mechanism: A retrospective cohort study

**DOI:** 10.3389/fphys.2022.958033

**Published:** 2022-12-14

**Authors:** Yifat Fundoiano-Hershcovitz, David L. Horwitz, Candy Tawil, Oded Cohen, Pavel Goldstein

**Affiliations:** ^1^ Dario Health, Caesarea, Israel; ^2^ DLH Biomedical Consulting, Las Vegas, NV, United States; ^3^ Integrative Pain Laboratory (iPainLab), School of Public Health, University of Haifa, Haifa, Israel

**Keywords:** digital therapeutics, pain biofeedback, digital biofeedback, pain management, posture, back pain, pain mechanism

## Abstract

**Introduction:** Back pain is an extremely common symptom experienced by people of all ages and the number one cause of disability worldwide. ^2^ Poor posture has been identified as one of the factors leading to back pain. Digital biofeedback technology demonstrates the promising therapeutic ability in pain management through posture training. One common goal of such an approach is to increase users’ posture awareness with associated movement correction. However, we lack a deep understanding of the biofeedback therapeutic mechanisms and the temporal dynamics of efficacy.

**Objective:** This study investigates the temporal dynamics of the biofeedback learning process and associated outcomes in daily life settings, testing the mechanism of the biofeedback-associated pain reduction.

**Methods:** This retrospective real-world evidence study followed 981 users who used the UpRight posture biofeedback platform. Piecewise mixed models were used for modeling the two-stage trajectory of pain levels, perceived posture quality, and weekly training duration following an 8-week biofeedback training. Also, the mediation effect of perceived posture quality on the analgesic effect of training duration was tested using Monte Carlo simulations based on lagged effect mixed models.

**Results:** The analysis revealed significant pain level reduction (*p* <.0001) and posture quality improvement (*p* <.0001) during the first 4 weeks of the training, maintaining similar pain levels and perceived posture quality during the next 4 weeks. In addition, weekly training duration demonstrated an increase during the first 3 weeks (*p* <.001) and decreased during the next 5 weeks (*p* <.001). Moreover, training duration predicted following-week perceived posture quality (*p* <.001) and in turn perceived posture quality predicted following-week pain (*p* <.001) (*p* = 0.30). Finally, perceived posture quality mediated the effect of weekly training duration on the pain levels in 2 weeks (*p* <.0001).

**Conclusion**: Our findings provide a better understanding of the therapeutic dynamic during digital biofeedback intervention targeting pain, modeling the associated two-stage process. Moreover, the study sheds light on the biofeedback mechanism and may assist in developing a better therapeutic approach targeting perceived posture quality.

## Introduction

Overall, back pain is an extremely common symptom experienced by people of all ages (Hartvigsen et al., 2018). It is the most frequent cause of chronic pain with a prevalence of around 18.3% worldwide (Vos et al., 2016). The (lower) back is the most common site for chronic pain, with a lifetime prevalence of 30–40% (Sielski et al., 2017), which has annual costs in the hundreds of billions of dollars in the United States (Kotlarz et al., 2009), and the associated pain contributes to the opioid epidemic (Ringwalt et al., 2014; Reuben et al., 2015). Back pain can result from several known and unknown abnormalities or diseases. Most cases of back pain are mechanical or nonorganic and are not caused by serious conditions, such as inflammatory arthritis, infection, or fracture (Vos et al., 2016; Casiano et al., 2021). Unfortunately, a precise pathoanatomical diagnosis cannot be given in about 80% of low back pain (LBP) patients (Foster et al., 2018), who are consequently labeled “nonspecific LBP” (Nijs et al., 2015) and whose treatment focuses mostly on reducing pain symptoms.

Nonpharmacologic approaches, including exercise, psychoeducation, and behavioral interventions, are universally recommended as the first-line treatment for the majority of chronic musculoskeletal conditions (Qaseem et al., 2017). Moreover, it can achieve similar outcomes to surgery with a reduced cost and lower risk [8, 9].

Postural control refers to building up posture against gravity and ensuring that balance is maintained. Correct posture is considered to maintain the natural curve of the spine in the human body and prevent damage or progressive deformation in all positions, including standing, lying down, and sitting (Kim et al., 2015).

Incorrect posture may have a negative effect on the spine (Wong et al., 2009; Alamin et al., 2018; Kwon et al., 2018). Previous studies have suggested a relationship between sagittal spinal malalignment and LBP (Hira et al., 2021). Sagittal curvature of the spine and pelvis stabilize each other to maintain a stable posture. Additionally, it was shown that a positive sagittal balance was significantly related to clinical symptoms in patients with adult spinal deformity (Glassman et al., 2005). When the sagittal alignment is anomalous, more energy is required to maintain balance without external support (Hira et al., 2021). Various adverse effects related to prolonged sitting or standing behaviors with incorrect posture were examined over longer time domains of 12 or 24 months. Cases of exhaustion during a working day, job satisfaction, hypertension, and low back pain among office workers were reported (Andersen et al., 2007; Waters and Dick, 2015; Daneshmandi et al., 2017). Poor posture is a known cause of low back pain; however, there is limited knowledge about the mechanisms involved (Hasegawa et al., 2018).

Postural awareness is basically defined as the individual’s mindful awareness of body posture that is mainly based on proprioceptive feedback from the body periphery to the central nervous system. Though the links between posture and pain are highly complex, studies have shown that improving habitual postural patterns might lead to improvements in musculoskeletal pain conditions and prevent further deterioration (Kent et al., 2015; Wälti et al., 2015; Cramer et al., 2018). Good posture and back support are critical to reducing the incidence of back pain. One approach to achieving better posture is choosing ergonomic office equipment that often provide better support and may be more comfortable for the patient or taking breaks from prolonged sitting/standing (John Schubbe et al., 2022). There are exercises developed by professional physiotherapists that help alleviate muscle tension caused by poor sitting or standing habits (Harvard Health, 2015; NHS, 2022).

The use of biofeedback has been offered in the past as an instrument for training that enables an individual to learn how to change physiological activity or behavior for the purposes of improving performance (Thanathornwong and Suebnukarn, 2019). Previous studies examined the effectiveness of postural biofeedback added to a conventional physiotherapy treatment for chronic low back pain (Sielski et al., 2017). Postural training with vibrational biofeedback was shown to reduce pain perception in the experience of LBP, the discomfort level of LBP, and LBP-related work interference rates (Park et al., 2018).

Interventions *via* the digital health platform have the potential to improve traditional care outcomes for chronic musculoskeletal pain by increasing patient engagement. Practically, it can better enable patients to take a proactive role in their therapy and learn to self-manage their pain symptoms (Bartys et al., 2017; Auerbach, 2019). In addition, patient persistence to seek surgical treatment is shown to decrease following participation in a digital health program (Cronström et al., 2019).

Eventually, the development and utilization of digital health interventions in a therapeutic capacity for musculoskeletal conditions need to work toward reducing the burden of musculoskeletal-related disability (Hewitt et al., 2020). An easy to wear and comfortable system allowing for digital signals suitable for evaluating “poor” or “good” posture may promote self-awareness of individuals’ behaviors as a means of motivating improvement and taking personal responsibility for their health (Giansanti et al., 2009; Simpson et al., 2019).

Overall validation studies for multiple systems of wearable posture monitoring have been favorable, although clinical integration of these systems has not yet emerged on a larger scale (McCullagh et al., 2016; Simpson et al., 2019). More investigation of the value of postural training using wearable biofeedback technology in the digital therapeutic management of back pain is required (Park et al., 2018). Limited research has been focused on reducing disability by improving exercise adherence in patients with back pain using a smartphone-based intervention, and there is still a lack of evidence for the efficacy of mobile health embedded in care in large-scale scientific analysis (Chhabra et al., 2018; Priebe et al., 2020). In addition, the temporal dynamics of analgesia associated with digital therapeutic technology is still not clear. Importantly, although the literature suggests that supportive techniques are effective and widely used, little is known about the mechanisms underlying their effect on therapy and pain (Leibovich et al., 2020). Previous research has demonstrated the ability of posture biofeedback technology to improve posture (Mathie et al., 2004; Park et al., 2018). Other studies have shown an association between perceived posture quality and pain levels (Kent et al., 2015; Wälti et al., 2015; Cramer et al., 2018). However, perceived posture quality has never been tested as a potential source for controlling posture biofeedback in alleviating pain.

This study leverages a retrospective analysis of a home-use posture biofeedback trainer with full data capture in a supporting mobile app among people suffering from pain. The purpose of this retrospective study was 1) to evaluate the biofeedback training dynamics and its efficacy on pain and perceived posture quality in patients suffering from back pain and 2) to test the mediating role of perceived posture quality in the effect of posture biofeedback on pain levels. Of note, previous studies suggested that changes in posture and pain outcomes appear to have the following two phases: an initial improvement over the first 4 weeks, followed by a longer-term sustained period (Correia et al., 2018; Park et al., 2018; Bailey et al., 2020).

We hypothesized that during the first 4 weeks of using a self-management app and training posture would be associated with reduced pain levels. By modeling the two-phase trajectory process, we expected to show the improvement to persist over an 8-week period. The present study aims to investigate the proposed mediation model, according to which posture training and improvement may fulfill the role of a mechanism of change that enables to achieve better pain reduction outcomes.

## Methods

### Users

This is a retrospective cohort study that uses a posture biofeedback digital platform with follow-up data collected between 2018 and 2020 (UpRight by Dario Health). The data were collected from the UpRight users during their actual biofeedback usage in the period of the first 8 weeks. The analysis included 981 users out of 6,098, who used the UpRight platform between 2018 and 2020. The inclusion criteria were as follows: high pain levels (>6) at the first assessment and at least two self-reported assessments of pain levels and posture quality during the first 8 weeks of biofeedback training sessions. Based on our pilot study, users who train less than 6 h a week are not engaged enough in the process and do not show the same linear grade of clinical improvement. For this reason, the users with less than six weekly training hours were removed from the main analysis and used as a “nonintensive training” group for sensitivity analysis (see below). The users’ average age was 40.8 (SD = 22.7) years, with an average height of 171 cm (9.90) and a weight of 71.8 kg (16.1). A part of 58.6% of the users was women. Ethical and Independent Review Services (Ethical and Independent Review Services, 2020), a professional review board, issued the institutional review board exemption for this study (21,048–01#).

### Platform

**FIGURE 1:**
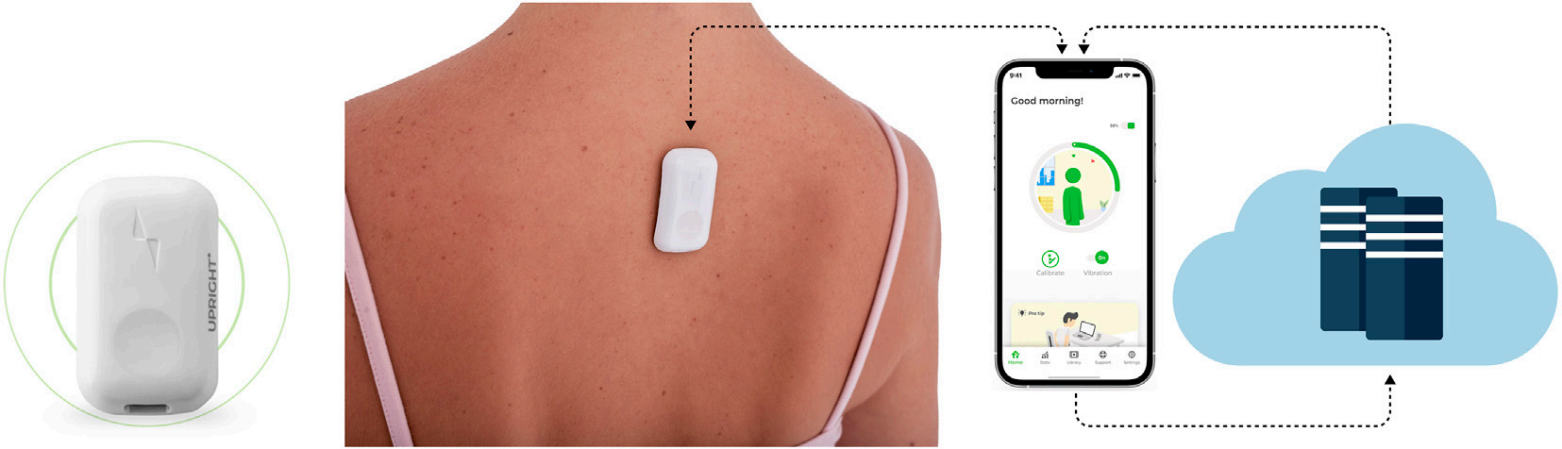
Digital biofeedback technology. The device is located on the upper back, and the app screens display upright and slouching positions based on sensor assessment.

This study utilized the postural biofeedback tactile feedback wearable device (UpRight by DarioHealth). The UpRight device is programmed to vibrate when slouching postures are detected (based upon changes in tilt and curvature of the spine) and alert users of their change in body position. The device is a postural training device that uses a triaxial accelerometer to set target posture and monitors body position to provide a vibration stimulus alert when good body posture is not being maintained. In a slouch position, each vibration cycle includes two vibrations and a break of 10 s, which is repeated continuously until a proper posture is achieved. The UpRight device control circuit was built using the following components: a system on chip based on a 32-bit ARM Cortex-M4F processor, a BLE transceiver operating at the 2.4 GHz frequency band for wireless data transmission, a 3.7 V lithium polymer battery as a power supply, and a motor driver for feedback. A transceiver unit was interfaced with the mobile application wirelessly to transmit the posture feedback. The motor driver drives an ERM (eccentric rotating mass) vibration motor. The base resonant frequency of the ERM motor is approximately 250 Hz. Moreover, there are eight types of vibration patterns that are available in the app for the user’s choice and preference. The difference between the patterns is their duty cycles and modulated PWM (pulse width modulation) frequencies. Visual feedback is available through the cell phone screen (Figure 1). The device is removed from the holder, placed in the middle upper back under the shirt, and directly connected *via* Bluetooth into a cell phone, effectively converting the cell phone into the display screen for the device. The users were instructed to attach the UpRight device in their upper back by the instructions provided in the UpRight app. The device allows the user to define the sensitivity for posture indication (red range indicator) varying between strict and relaxed using a six-level bar (1, strict; 6, relaxed, 3° intervals) determined in reference to the calibration state. Connecting the meter directly to the phone ensures real-time data capture during a posture training session. In a “calibration” screen, the user calibrates upright to his back. The user is instructed to sit upright and stay still, once ready to press the “I’m upright” button. The device is recording the upright position of the user. The user may define the delay time for vibrotactile feedback for tilt angle with reference to calibration. The delay time range is 5, 15, 30, or 60 s. When the user starts logging in to the app and using the device, a default recommendation is presented to the user to complete the amount of time spent in an upright position in a day (uptime goal). The first daily goal is 60 min, which increases gradually throughout the program according to the user’s progress (the upper limit of daily goals is 500 min).

### Measures

In this retrospective cohort study, users with high pain levels used a biofeedback posture device to improve their posture. Training duration—the time the user used the posture biofeedback (in hours)—was recorded. After a session, the user was asked to rate the level of back pain using the numerical rating scale (NRS) on a 0–10 scale (Haefeli and Elfering, 2006) (0, no pain; 10, extreme pain) and his posture on a 0–10 scale (0, mostly slouched; 10, mostly upright) in data entry screens. A numerical scale was used to enable the user to directly read the pain intensity from the screen. This graphic rating scale was found to correlate positively with other self-reporting measures of pain intensity (Haefeli and Elfering, 2006). The weekly average pain level, which was defined as the mean of all of the users’ pain ratings taken over a 7-day interval, was used as a core outcome metric. The weekly average posture level, which was defined as the mean of all of the users’ posture ratings taken over a 7-day interval, was used as a mediator. Total training duration was averaged for the same 7-day intervals using the minute time scale. In addition, users’ demographic variables such as gender, weight, height, and age were collected. The UpRight device is connected to the mobile device directly. The mobile app transmits the data to the public cloud platform, where the data are stored in PostgreSQL and BigQuery. All data were processed and stored in compliance with GCP and AWS standards. All data were anonymized before extraction for this study.

### Sensitivity analysis

Additional sensitivity analysis ensured that age, gender, weight, and height did not modify the findings of the mediation analysis. To compensate for the absence of the control group, we conducted an additional sensitivity analysis in the following way: all of the participants were assigned to “intensive training” or “nonintensive training” groups if, during the first week, they used the biofeedback for six or more hours or less than 6 h, respectively. The two groups are not associated with age, gender, height, weight, and BMI (all P’s > 0.1).

### Analytical approach

Statistical analysis consists of two parts: 1) nonlinear time trajectory analysis of pain levels, perceived posture quality, and weekly training duration and 2) testing the mediation effect of perceived posture quality on the analgesic effect of training duration. The first part is focused on modeling weekly pain levels, perceived posture quality, and weekly training duration throughout the users’ initial 8 weeks of using UpRight biofeedback. The second analysis is designed to test the mediation effect of perceived posture quality in the effect of training duration on pain levels using lagged effects (see below).

### Nonlinear trajectory analysis

Traditional linear longitudinal models assume a single growth pattern in an outcome across time. Sometimes, this assumption does not fit the empirical data. In contrast, piecewise-based mixed-effect models allow flexibility in the modeling of trajectories across time (Fundoiano-Hershcovitz et al., 2021). Herein, a mixed piecewise model is used as a framework for analyzing pain levels and perceived posture quality in two segments with the R package lme4, namely, 1) training-related improvement (1–4 weeks) and 2) the training effect maintenance (5–8 weeks). A similar analysis was also conducted for the weekly training duration, but this time the two parts were defined for 1–3 weeks and 4–8 weeks, correspondingly. The piecewise approach allows for the modeling of different linear trends over the different regions, providing an opportunity to model curvilinear changes in the weekly pain level, perceived posture quality, and weekly training duration as an integral process. Following previous research (Correia et al., 2018), the piecewise cutoff point for modeling the outcome slopes was chosen at 5 weeks, assuming a shift in the time trajectory after 4 weeks of using UpRight posture biofeedback. The model included person-based random slopes for both periods (1–4 and 4–8 weeks).

The model formulation is as follows:

*Level 1*: Yij=β0j+β1j*time1+β2j*time2+Rij

*Level 2*: β1j=γ10+U1j,β2j=γ20+U2j ,

where i represents the week, j represents a user, and time1 and time2 are the piecewise dummy parameters for weeks 1–4 and 4–8, respectively, and β1/β2 is their slope. R represents the model general residuals, and U1\U2 represents random slopes for the model slopes, while γ10 and γ20 are the corresponding fixed effects.

### Mediation analysis

The mediation model relies on the assumption of casual associations between the predictor to mediator and the mediator to the outcome. Practically, this is hardly achievable. Even the analysis based on a randomized controlled trial may ensure causality just for one of the model paths. As a solution, lagged analysis was suggested to ensure temporal order while testing the association of interest (Wu et al., 2018). A lag of one time unit (1 week) is enough to create temporal order, assuming that the predictor measured at a certain week is associated with the following-week mediator that in turn will be associated with the model outcome measured 1 week later.

We applied the mixed modeling framework for mediation testing using a similar statistical framework. To examine a mediation model, we conducted a series of analyses in which the following were tested: 1) the association between training duration (week t) and the following week’s (t+1) k perceived posture quality and 2) the association between perceived posture quality (week t+1) and users’ pain ratings in the following week (t+2). Thus, the outcome measure for the mediation analysis was the users’ weekly aggregated pain rating 2-week lagged, while the mediator was users’ weekly aggregated perceived posture quality rating 1-week lagged. Technically, we applied the following two models: a) to test the 1-week-lagged association between training duration and following perceived posture quality and b) to test the association between 1-week-lagged perceived posture quality and 2-week-lagged pain ratings, conditioned on the effect of the training duration. Finally, the mediation effect was defined as a*b, and statistical inferences were made based on the approach described earlier. The mediation model was tested using a quasi-Bayesian–Monte Carlo method with 5,000 simulations, and White’s heteroskedasticity-consistent estimator for the covariance matrix was used to overcome the violation of the residual homoscedasticity assumption (Zeileis, 2006; Tingley et al., 2014). Finally, the mediation model was tested where the group differences in pain rating were mediated by perceived posture quality (group→perceived posture quality→pain levels). Taking advantage of the lagged analysis and sensitivity analysis, the current study has the quality of the quasi-experimental design and testing quasi-casual, thus boosting the validity of the performed mediation analysis.

## Results

### Nonlinear analysis of pain levels, perceived posture quality, and training duration trajectories over time

**FIGURE 2:**
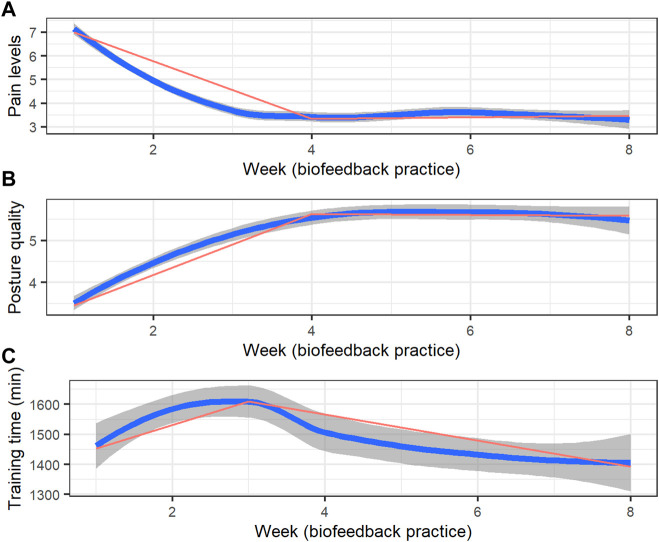
Training trajectories (weekly aggregates over a 7-day interval) of **(A)** pain ratings, **(B)** perceived posture quality, and **(C)** training duration (in hours). The blue line represents locally weighted smoothed weekly averages with 95% confidence intervals (the dark grey area surrounding each curve), and the red line represents the applied piecewise model.

Table 1 presents the descriptive statistics of the sample. The nonintensive training group shows a similar distribution to the intensive training group based on the collected data. Figure 2 presents the weekly aggregates of pain levels, perceived posture quality, and training duration trajectories. Piecewise mixed model analysis revealed a significant pain level reduction (B = −0.80, 95% CI −0.81 to −0.79, *p* <.0001; 50% reduction, R^2^ = 0.09, 3.84 points) and perceived quality improvement (B = 0.72, 95% CI 0.63 to 0.82, *p* <.0001; R^2^ = 0.10) during the first 4 weeks of the training, maintaining similar pain levels (B = 0.06, 95% CI −0.03 to 0.14, *p* = 0.20) and perceived posture quality (B = −0.02, 95% CI −0.16 to 0.08, *p* = 0.68) during the next 4 weeks. Lastly, weekly training duration demonstrated a significant increase during the first 3 weeks (B = 47.19, 95% CI 1.25 to 95.40, *p* = 0.04, R^2^ = 0.02) and significantly decreased during the next 5 weeks (B = -85.75, 95% CI -106.09 to −65.28, *p* <.001).

**TABLE 1:** Descriptive statistics of the sample.

	Nonintensive training (N = 656)	Intensive training (N = 981)	Overall
Gender			
Female	63.9%	59.1%	61.5%
Male	36.1%	40.9%	38.5%
Age			
Mean (SD)	38.6 (38.8)	39.8 (12.7)	39.2 (29.0)
BMI			
Mean (SD)	24.6 (4.85)	24.5 (4.52)	24.6 (4.69)
Training duration (min)			
Mean (SD)	224 (189)	1,350 (785)	819 (812)

### Testing the mediating role of perceived posture quality in the effect of training duration on pain levels

**FIGURE 3:**
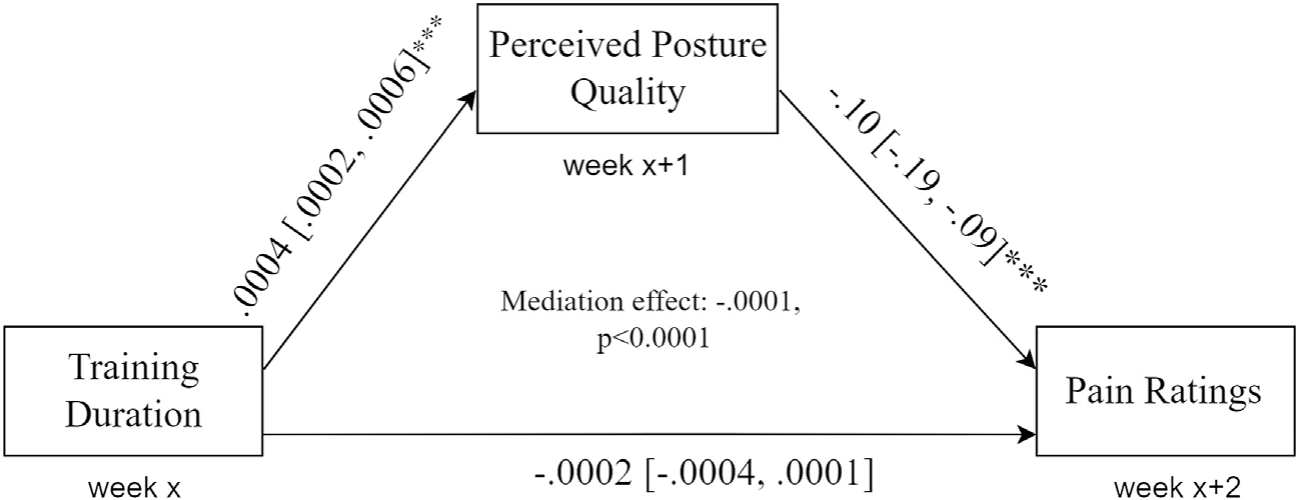
Mediation model. Training duration is significantly associated with the following-week perceived posture quality, which in turn predicts next week’s pain levels. Perceived posture quality mediates the association between training duration and pain levels.

The direct association between training duration and pain levels was significant (B = −0.0002, 95% CI -0.0003 to -0.0001, *p* <.001, R^2^ = 0.1). Training duration significantly predicts following-week perceived posture quality (B = 0.0004, 95% CI 0.0002 to 0.0006, *p* <.00, R^2^ = 0.12) and, in turn, perceived posture quality significantly predicts following-week pain (B = −0.10, 95% CI -0.019 to -0.009, *p* <.001, R^2^ = 0.09), adjusting for previous week training duration quality that was not significant in this model (B = −0.0002, 95% CI -0.0004 to 0.0001, *p* = 0.30). Finally, perceived posture quality significantly mediated the effect of weekly training duration on the pain levels in 2 weeks (M = −0.0001, *p* <.0001) (Figure 3).

### Sensitivity analysis of the mediation effect

The sensitivity analysis conducted to establish a control group assigned the participants to an “intensive training group” or a “nonintensive training group” and tested the mediation model, where group differences in pain rating are mediated by perceived posture quality (group→perceived posture quality→pain levels). The “intensive training” group demonstrated significantly higher perceived posture quality (B = 0.35, t = 3.23, *p* = 0.001, 0.14–0.57). Also, perceived posture quality showed a significant negative association with pain rating (B = -0.08, t = -4.49, *p* = 0.001, -0.12 to −0.04), controlling for the group assignment that was not associated with pain ratings in this model (B = 0.002, t = 0.02, *p* = 0.98, −0.21–0.21). Finally, perceived posture quality significantly mediated the effect of the group (“intensive training” vs. “nonintensive training”) on the pain levels (M = −0.03, *p* < 0.0001). The effect of the group on pain levels was saturated by the perceived posture quality, supporting the full mediation effect of the perceived posture quality.

## Discussion

### Principal results

This study presents a real-world analysis of the posture biofeedback efficacy and the temporal resolution of its effect, as well as testing a potential mechanism of the associated analgesia. More specifically, we found statistically significant and clinically meaningful pain reductions associated with the biofeedback training. Then, 62% of the users reported pain reduction beyond the minimal clinically important difference (two points on the NRS), while 76% showed some pain reduction. Interestingly, 60% of the users demonstrated pain reduction beyond the minimal clinically important difference already after 4 weeks of biofeedback training, confirming the findings of our two-slope dynamic model. Our findings confirmed two distinct phases in biofeedback training: 1) a rapid improvement phase lasting 4 weeks, associated with pain reduction and increased perceived posture quality, followed by 2) a maintenance phase lasting for the next 4 weeks, associated with no changes in pain and perceived posture quality. In addition, weekly training duration increased during the initial 3 weeks and decreased during the following 5 weeks. This study showed that the utilization of a piecewise mixed model statistical framework appears to be an appropriate base model to describe nonlinear fluctuations in pain levels, perceived posture quality, and training duration.

Finally, we demonstrated that training duration predicts changes in the following-week perceived posture quality, which in turn predicts users’ pain ratings in the following week. Perceived posture quality significantly mediated the lagged association between training duration and pain levels.

### Comparison to prior work

Consistent with the literature, we found that users of digital biofeedback experienced the most change in their first few weeks of use and generated comparable improvements that are in line with the recent trends of healthcare delivery toward home-based care (Kramer et al., 2003; Correia et al., 2018; Bailey et al., 2020).

Biofeedback treatment can lead to improvements on various pain-related outcomes in the short and long terms (Sielski et al., 2017).

The precise physiological mechanism of posture biofeedback is still not clear. Our findings are in line with previous studies that showed that increasing training hours and providing feedback regarding faulty static posture for 3 weeks was associated with improved perception of spinal posture (Park et al., 2018).

One line of the research states that in order to obtain good posture, inactive individuals need to build up the proper muscle balance, strength, and endurance to stabilize the spine (Budhrani-Shani et al., 2016; Canadian Chiropractic Association - Association chiropratique canadienne, 2018). However, it was also reported that postural training with biofeedback may help to reduce stress, fatigue, and psychological disorders that are associated with faulty spinal posture (Park et al., 2018). Indeed, recent studies have shown that an increase in strength over the first few weeks of resistance training has a neural component, demonstrating brain neuroplastic changes associated with pain (Pearcey et al., 2021). When the brain experiences pain over and over, neural pathways get strengthened and sensitized (Woolf, 2011; Baliki and Apkarian, 2015).

Recently, back pain was associated with neural activity in the brain regions responsible for emotional processing rather than typical nociceptive processing (Hashmi et al., 2013). However, due to the data limitations, we cannot ensure that all of the users had primary chronic pain. Thus, other types of chronic pain can be involved in other potential therapeutic mechanisms. Moreover, back pain is related to emotional perception in the body (Chojnacka-Szawłowska et al., 2019; Goldstein et al., 2020), while perceived body posture quality can be driven by changes in subjective perception of one’s posture. The links between posture and pain are complex; however, studies have shown that improving habitual postural patterns might lead to improvements in musculoskeletal pain conditions and prevent further deterioration (Kent et al., 2015; Wälti et al., 2015; Cramer et al., 2018). Changing patterns of posture using motion-sensor biofeedback in people with LBP resulted in a reduction in pain and activity limitation when compared with guideline-based medical or physiotherapy care (Kent et al., 2015).

Previous research studies have shown a complex interaction between pain and body perception disturbances (Lewis et al., 2007). Clinical evidence supports the use of treatments that target cortical areas, which may reduce body perception disturbances and pain (Moseley, 2006). A greater knowledge of body posture perception may provide valuable insights into the mechanisms of the central neural system associated with analgesia. There is convincing evidence demonstrating links between poor or disrupted awareness of sensory information or interoceptive awareness and difficulties with physical and emotional regulation (Borg et al., 2018).

In line with the health behavior change theory, it posits that new health behaviors emerge when people gain both knowledge and awareness (Lawson and Flocke, 2009; Lustria et al., 2013; van der Laan et al., 2017). We suggest that the time of training one’s posture using biofeedback is a prime opportunity for reinforcing knowledge and executing behavioral change that further affect pain level. In other words, by posture training, people with pain pay attention to their positioning and movement and turn it into a moment of reflection on their actions preceding that training. This time period of focused awareness-building may be a key piece in launching a virtuous process of improved future health behaviors (Moseley, 2004).

In this study, it was demonstrated that users’ pain ratings changed nonlinearly over time. Previous reviews of randomized clinical trials for low back pain showed comparable changes. In line with our findings, they have also demonstrated musculoskeletal pain reduction in the initial period of 4 weeks (Artus et al., 2010; Correia et al., 2018; Bailey et al., 2020).

One of the objectives of the digital biofeedback technology concept is to prevent patients at risk from developing chronic pain. To boost the efficacy of the preventive intervention, we need a better understanding of its mechanism. Through the retrospective analysis of the existing data collected through the app, it became evident that perceived posture quality is potentially one of these mechanisms.

Mobile technology use was previously shown to effectively promote certain desired behaviors including physical activity. However, there is a lack of studies that focused on reducing pain or disability by improving training adherence and behavioral change in patients suffering from back pain using a smartphone-based intervention (Evans et al., 2012; Chhabra et al., 2018).

Based on our findings of the mediation model created on the lagged association between training duration, perceived posture quality, and pain levels, we suggest that perceived posture quality is a potential mechanism of posture training-related analgesia.

Behavioral changes are mostly nonlinear in time and modeled here in two linear trajectories (Glasgow et al., 2012; Shan et al., 2019; Bailey et al., 2020; Fundoiano-Hershcovitz et al., 2021). Herein, we show it as a single process applying the piecewise model. Piecewise-based mixed-effect models allow for flexibility in the modeling of variable change trajectories across time. Herein, a mixed piecewise model assessed differences in the weekly average pain and posture levels in two segments (Kohli et al., 2018). Future research should focus on personalizing biofeedback technology by identifying target populations and optimizing training programs.

### Limitations

Several limitations should be mentioned. First, as in all studies involving retrospective real-world data, the casual interference here is limited due to the nonexperimental design of the study and the absence of the control group. Additionally, real-world data source and type of data used may limit the generalizability of the results and of the endpoints and only evaluate association and not causality. To increase the validity of the proposed mediation model, we created temporal lags for the variables involved in the mediation analysis for the quasi-causal inference.

Second, using a digital therapeutic tool that allows for the recruitment and management of an increased number of subjects for the study limits the number of applied assessments. Third, in this real-world data analysis, the time scale was designed to reflect weekly interval change over an 8-week period. However, the relationships of interest in this study could be potentially investigated in different scales emphasizing daily fluctuations. Owing to the difficulty in tracking daily changes in digital engagement in real-world studies, most studies focus on weekly or monthly fluctuations. Investigating fine-grained measurements with microintervals for tagging would certainly contribute to the literature (Wagner et al., 2017). Fourth, demographic data were limited. While age, gender, weight, and height did not affect the findings, uncontrolled demographic biases (e.g., race) might have been present from other demographic factors. Additionally, the exact pain condition, other treatments, or medications used are missing, and the exclusion of the nonresponders from the main analysis may bias the findings. Fifth, although the NRS is considered the “gold standard” for the assessment of clinical and suprathreshold experimental pain, more comprehensive assessments may improve the accuracy of the collected subjective information (e.g., pain interference, fear of pain, and PROMIS pain intensity).

## Conclusion

It appears highly likely that posture biofeedback may serve as a potential tool for the associated pain management. We found statistically significant pain reduction associated with the biofeedback training, focusing on the temporal resolution of the associated effects. Finally, we have demonstrated that perceived posture quality may serve as a potential mechanism associated with posture training analgesia.

Future work investigating posture mechanisms such as motion capture and electromyography on a larger scale is required. In addition, longer time intervals of 3–6 months of postural training and follow-up may increase the reliability and validity of the clinical efficacy and associated mechanisms.

## Data Availability

The raw data supporting the conclusions of this article will be made available by the authors, without undue reservation.

## Abbreviations

LBP, low back pain; NRS, numerical rating scale.
